# The role of therapeutic optimism in recruitment to a clinical trial in a peripartum setting: balancing hope and uncertainty

**DOI:** 10.1186/s13063-016-1394-1

**Published:** 2016-06-01

**Authors:** Nina Hallowell, Claire Snowdon, Susan Morrow, Jane E. Norman, Fiona C. Denison, Julia Lawton

**Affiliations:** Ethox Centre, Nuffield Department of Population Health, University of Oxford, Oxford, OX3 7LF UK; Department of Medical Statistics, London School of Hygiene and Tropical Medicine, London, UK; Centre for Population Health Sciences, University of Edinburgh, Edinburgh, UK; MRC Centre for Reproductive Health, University of Edinburgh, Edinburgh, UK

**Keywords:** Therapeutic optimism, Uncertainty, Hope, Trial recruitment, Individual equipoise, Qualitative interviews

## Abstract

**Background:**

Hope has therapeutic value because it enables people to cope with uncertainty about their future health. Indeed, hope, or therapeutic optimism (TO), is seen as an essential aspect of the provision and experience of medical care. The role of TO in clinical research has been briefly discussed, but the concept, and whether it can be transferred from care to research and from patients to clinicians, has not been fully investigated. The role played by TO in research emerged during interviews with staff involved in a peripartum trial. This paper unpacks the concept of TO in this setting and considers the role it may play in the wider delivery of clinical trials.

**Methods:**

The Got-it trial is a UK-based, randomised placebo-controlled trial that investigates the use of sublingual glyceryl trinitrate (GTN) spray to treat retained placenta. Qualitative data were collected in open-ended interviews with obstetricians, research and clinical midwives (*n* =27) involved in trial recruitment. Data were analysed using the method of constant comparison.

**Results:**

TO influenced staff engagement with Got-it at different points in the trial and in different ways. Prior knowledge of, and familiarity with, GTN meant that from the outset staff perceived the trial as low risk. TO facilitated staff involvement in the trial; staff who already understood GTN’s effects were optimistic that it would work, and staff collaborated because they hoped that the trial would address what they identified as an important clinical need. TO could fluctuate over the course of the trial, and was sustained or undermined by unofficial observation of clinical outcomes and speculations about treatment allocation. Thus, TO appeared to be influenced by key situational factors: prior knowledge and experience, clinical need and observed participant outcomes.

**Conclusions:**

Situational TO plays a role in facilitating staff engagement with clinical research. TO may affect trial recruitment by enabling staff to sustain the levels of uncertainty, or individual equipoise, necessary to collaborate with research while also responding to patients’ clinical needs. Staff may benefit from training to deal with fluctuations in TO.

**Trial registration:**

ISCRTN88609453. Registered on 26 March 2014.

## Background

This paper is based upon data generated in interviews with healthcare professionals as part of a qualitative evaluation of a UK peripartum randomised placebo-controlled trial (RCT), the Got-it trial, which looked at the use of glyceryl trinitrate (GTN) for the delivery of retained placenta (see Table 1). The Got-it trial contained an inbuilt pilot phase, and certain recruitment targets had to be reached for the trial to continue. The aim of the qualitative research was to evaluate staffs’ and patients’ experiences of the trial’s recruitment procedures and consent pathway to see if these aspects of trial delivery could be improved prior to rollout of the main trial. This evaluation was included because the trial team were concerned that staff may find recruiting and consenting women challenging because the trial was taking place during the peripartum period, when women were in a vulnerable situation and required to make a decision about trial participation quickly. Thus, a significant proportion of the interviews with staff were spent exploring their broader views about, and understandings of, the trial, including its role and purpose, in addition to its organisation and their experiences of recruitment and consenting individual women. These questions sought to determine what interviewees thought were the barriers and facilitators to recruitment and how these might be overcome or emphasised in the main trial, so that staff could be best supported to undertake recruitment. When answering these questions, staff reflected at length upon their hopes and uncertainties regarding the Got-it trial. While we had expected that staff would be primarily concerned with the organisational issues associated with trial recruitment and delivery, as the interviews progressed it became clear that a sense of optimism about the trial was critical to staffs’ on-going commitment to, and involvement with, the trial and thus fundamental to ensuring recruitment to Got-it. The idea of therapeutic optimism (TO) in the context of clinical research has been briefly discussed in the literature, but the role it might play in the delivery of clinical trials is poorly defined, as is the concept itself. Using empirical data collected in interviews with staff, this paper will expand the concept of TO by looking at the role it plays for staff recruiting to the Got-it trial.

**Table 1:** The Got-it trial

“The Got-it trial is a randomised placebo controlled double blind pragmatic UK-wide RCT involving women who have a retained placenta (RP) recruited from delivery wards in UK maternity hospitals. RP is a major cause of postpartum haemorrhage and affects around 2 % of vaginal deliveries in the UK. It is diagnosed when the placenta is not delivered within 30 minutes following active management or 60 minutes after physiological followed by active management of the third stage of labour after delivery of the baby [26]. Although some placentas can still be delivered vaginally after a RP is diagnosed, the chance of this happening is low and decreases the longer the placenta remains in situ. The definitive management of RP is, therefore, manual removal of the placenta which is a surgical procedure requiring trained personnel and an operating theatre. The aim of the Got-it trial is to determine whether use of glyceryl trinitrate (GTN) spray, as compared to a placebo, can facilitate delivery of the placenta without having to undertake manual or surgical delivery in theatre. GTN is a drug that was originally developed for the prevention and relief of angina attacks. Its side-effects include headache, dizziness, flushing/feeling hot, a drop in blood pressure or a rise in pulse. In the clinical context of RP, it could also affect blood loss due to its primary mode of action as a muscle relaxant. For the trial’s inclusion and exclusion criteria see Lawton et al. [17].
The trial comprises an internal pilot followed by a substantive RCT. The pilot commenced in October 2014 and involved eight sites that entered the pilot in a staggered way. During the pilot, once a diagnosis of RP had been made, a delegated and trained member of the clinical or research team approached potential recruits. These women were given written information in the form of a one page summary leaflet accompanied by a detailed participant information sheet. Women were also given a verbal explanation of the trial that covered all the elements in the participant information sheet and consent form. Women who gave their consent were randomized to receive GTN or a placebo spray, which they self-administered under their tongue (two puffs). The placebo spray was designed to be identical in taste and appearance to GTN so neither participants nor staff could determine the outcome of randomization. Women who did not deliver their placentas within 15 minutes were taken to theatre for manual removal of the placenta under regional or general anaesthesia, with the method of anaesthetic being determined by the clinical team and being dependent on the urgency of need for placental delivery.”
Excerpts from Lawton et al. [17]

### The role of hope, therapeutic optimism and uncertainty in clinical research

The phenomenon of hope, expressed as therapeutic optimism, is an essential aspect of medical care.^1^ Hope is described within the medical and bioethics literature as having therapeutic value because it enables people to cope with uncertainty about their future health [1, 2]. Hope or TO can be defined as a positive, future-oriented [1] emotional state, which manifests as a desire for a particular healthcare outcome [3]. Martin [3] contends that hope also involves cognitive elements, insofar as it plays a role in framing individuals’ imaginative engagement with, and understanding and use of, information about desired outcomes. These features, he argues, may result in biased decision-making about medical treatment or research participation and thus render patients “vulnerable to exploitation” ([3], p. 52). For example, individuals’ desire for a cure can bias their perceptions of research outcomes and influence their interpretation of information about research participation [2, 4].

Research participation is frequently described as motivated by a number of cognitive biases or “mis”understandings that help individuals to deal with the uncertainty that lies at the heart of clinical research [5]. In a trial context, in addition to *therapeutic optimism* (*TO*), namely, hoping that one will benefit from trial participation [3, 6], these include *therapeutic misconception* (*TM*), or conceiving of research as needs-driven rather than hypothesis-driven, which is associated with holding the belief that the care offered in the trial is driven by personal need not scientific curiosity [7–10], and *therapeutic misestimation* (*TME*), or consistently (mis)estimating or (mis)calculating the risks and benefits of participation in one’s favour [4, 5]. It has been argued that all of these may have ethical consequences because they can influence participants’ decision-making and, therefore, their ability to give fully informed consent [2–4, 6, 8, 11–13].

Although TO, TM, and TME have been observed frequently in research participants [2, 9, 12, 13], the extent to which healthcare professionals subscribe to these biases is less well documented. Research suggests that clinical staff may be subject to TM [14], and TME and TO have been observed in staff involved in the delivery of phase 1 oncology trials [4]. Few researchers have looked at whether and in what ways TO influences staff outside of phase 1 trials, although a number of authors have suggested that researchers may capitalise on potential participants’ TO in their efforts to increase trial recruitment [3, 4, 6, 15].

### Characterising therapeutic optimism in clinical research

The literature suggests that hope and uncertainty exist in a complex relationship within clinical research. Clinical trials take place because there is uncertainty about the efficacy and utility of an intervention, but while uncertainty (or clinical equipoise) is a necessary feature of ethical research [16], researchers and research participants may find this unsettling and attempt to counterbalance their feelings of uncertainty with hope, and this may have ethical repercussions [4, 6].

The degree to which TO affects the ethicality of research has been discussed by Horng and Grady [5], who suggest that TO, or hope, is unavoidable when participating in clinical research. In contrast to TM or TME, they argue that TO — “personal optimism” or hoping for the best — is not ethically problematic, should be tolerated and even “actively preserved” ([5], p. 16), at least until it results in “misunderstanding” the risks and benefits of trial participation. However, Horng and Grady [5] are unclear about how misunderstanding can be distinguished from TO, and this, Jansen [6] argues, is because they do not specify the nature of TO, and also view it as a uni-dimensional phenomenon.

In her 2011 paper, Jansen [6] argues that what the literature describes as TO is, in fact, two contrasting phenomena. The first, *dispositional optimism,* is an individual character trait or disposition, which manifests as a generalised positive outlook on the world — always hoping for the best. The second, *situational optimism*, is focussed upon “particular events or activities” — hoping for the best in this situation. Situational optimism can be seen as an emotional response or reaction to a particular aspect of the environment and, because it is influenced by external factors, may be realistic or unrealistic. Dispositional optimism, on the other hand, is a way of interacting with the world, which, because it is internally generated, cannot be categorised as realistic or unrealistic. Jansen argues that these different types of optimism have differing ethical implications for clinical trials. For example, she suggests that research participants who are by nature optimistic, i.e. who exhibit dispositional optimism, are more likely to give valid informed consent than those whose optimism is influenced by external factors and who have unrealistic expectations about a particular outcome, i.e. display unrealistic situational optimism. The latter, she contends, are more vulnerable to external manipulation and prone to biased decision-making. Thus, for Jansen, dispositional optimism is seen as much less ethically problematic than situational optimism, which can be influenced by others and, in the case of unrealistic situational optimism, may invalidate informed consent by undermining individuals’ autonomy.

One of the problems with Jansen’s analysis of TO is that it does not specify how the different dimensions of TO are expressed, interact and potentially influence behaviour. But while her analysis may be less well defined than one might like, arguably in distinguishing different types of TO and examining their ethical significance, Jansen makes an important observation - namely, not all manifestations of TO are equal when it comes to their ethical implications. That said, following Jansen, it can be argued that if her conception of situational TO is to be used more widely, then we need to determine how situational TO becomes manifest and is sustained in practice.

To date, most of the discussion about TO in the context of clinical trials has dealt with research participants — focussing on how TO may bias their decision-making and invalidate their consent or how participants’ TO can be manipulated by researchers during the recruitment process. However, as noted above, it is not only patients or their proxies who may be optimistic about research interventions. As both Miller [4] and Martin [3] argue, clinical researchers may be optimistic about the outcomes of a clinical trial; they may hope for beneficial outcomes for their patients and this may influence their approach to research. Using staff accounts of their involvement in a peripartum RCT, this paper looks at how TO may influence staff participation in research. The analysis presented here suggests that, in certain circumstances, hope, expressed as *situational therapeutic optimism*, facilitates staff engagement with research and, therefore, may influence trial recruitment.

## Methods

### Study aims

The aim of this qualitative study was to explore staff’s and women’s experiences of, and views about, the information and consent pathway used in the pilot phase of the Got-it study (see Table 1). Full details of the main trial can be found on the International Standard Randomised Controlled Trials Number (ISRCTN) website.^2^ The analysis reported below focuses on staff members’ views and experiences of recruiting to the trial; data collected in interviews with women have been reported elsewhere [17].

### Study setting

Before describing our study, it is important to provide some context for the interpretation of our data. First, with regard to the trial itself, it must be noted that the study drug (sublingual glyceryl trinitrate spray, GTN) used in Got-it has a very short half-life (Table 1). This means that the primary outcome, namely, the delivery of the placenta, will occur within 15 minutes of administration of the trial intervention, thus enabling recruiting staff to directly observe the outcome of drug administration in their patients. In other words, in contrast to many trials where the primary endpoint may be spatially and temporally removed from the recruitment site, the link between intervention and outcome in Got-it is fairly tight, and thus, while the trial is blinded to staff (and research participants), the outcome for individual women is “directly” observable by those involved in trial recruitment.

Second, we feel it is important to provide some details of the clinical context in which the Got-it trial takes place. Because continuity of care is emphasised in birthing centres and labour wards, midwives (and doctors) often develop close (and intimate) relationships with the women in their care. If a woman has to go to theatre for a manual removal of the placenta, many of the important jobs that midwives have to do in the immediate postpartum period (i.e. initiating mother-baby bonding, skin-to-skin contact with baby and breast-feeding initiation) will be delayed, and in some cases the much valued continuity of care will be interrupted. Moreover, it is generally acknowledged that, while a manual removal of the placenta normally takes place under regional anaesthesia with the woman awake, it is an unpleasant and invasive procedure, particularly if it follows a labour that has involved the minimum of intervention and pain relief. Thus, arguably, birthing centre and labour ward staff have a vested interest in the Got-it intervention working, because if GTN works, it means that midwives can maintain their relationship with women, deliver their postnatal care more efficiently and potentially move the woman on to the postnatal ward more quickly.

### Ethical approval

REC approval was given by the Newcastle and North Tyneside 2 Research Ethics Committee (13/NE/0339). The study was also approved by the Medicines and Healthcare Products Regulatory Agency (MHRA), Eudract Number: 2013-003810-42. Written informed consent was obtained from participants for publication of their individual details and accompanying quotes in this manuscript. The consent forms are held by the authors and are available for review by the Editor-in-Chief.

### Recruitment

Staff members involved in recruitment to the pilot phase of the trial were sent invitation packs containing opt-in forms, information leaflets and consent forms. Staff were recruited from all of the eight pilot sites. Individual staff members were approached following their involvement in the recruitment of individual women, and we continued recruiting across the pilot sites until we had a sample that included staff with varying degrees of exposure to the trial. Recruitment continued until data saturation was achieved, that is, until no new themes were identified in new data. To safeguard confidentiality, all participants were allocated pseudonyms, and these are used below.

### Data collection and analysis

With one exception, data were collected during telephone interviews (40–90 minutes duration) conducted between November 2014 and May 2015. The interview topic guide was based upon the research questions and the literature, and included questions about: experiences of trial delivery — particularly recruitment and taking consent — perceptions and understanding of the trial design and the intervention (GTN) and general views of participation in research and its impact on the healthcare professional-patient relationship. The study was informed by the principles of grounded theory [18] and involved simultaneous data collection and analysis. All interviews were audio-recorded and transcribed. A conceptual framework for indexing and analysing data was developed using the method of constant comparison [19]; this enabled the identification of recurrent themes between and within interviews. Transcripts were read and coded by NH and JL, who discussed the emerging findings. NVivo9 (QSR International) software was used to facilitate data coding and retrieval.

## Results

### Interview participants

Of the 37 staff members who replied, 27 (73 %) were interviewed, including clinical midwives, research midwives and obstetricians (consultants, registrars, specialist trainees) (Table 2). As Table 2 indicates, the final sample had varying amounts of clinical and research experience.

**Table 2:** Participant characteristics

	*N* = 27	Percent
Obstetricians	10	37
Clinical midwives	6	22
Research midwives	11	41
Education		
Professional qualifications	1	4
Degree	26	96
Higher degree	5	19
Time in current post (years)		
0-2 years	9	41
2.5-5years	13	48
5.5-10 years	2	7
> 10 years	3	11
No previous research experience		
Research midwives	0	0
Obstetricians	4	15
Midwives	2	7

Staff were purposively sampled to reflect different trial experiences. Some sites had come on board at the start of the pilot and had been recruiting throughout the 7-month period, whereas others had only gone live during the last 2 months of the pilot phase. Length of time recruiting to the trial in the site plus the size of site meant that recruitment rates across the sites varied, and this was reflected in our sample, which included staff from sites where between 2–14 (mean 7.5) women had been recruited during the period when staff were interviewed. Some staff were interviewed relatively late in the pilot phase at their site, allowing them opportunity to reflect back on a range of trial experiences, whereas others were interviewed soon after the trial had gone live at their site and thus had less exposure to trial outcomes. Between 1–6 (mode 3) staff members were interviewed from each site.

When reflecting upon their experiences of trial recruitment, staff talked about their uncertainties and hopes for the trial. We will begin by describing the staffs’ uncertainty about the trial and then show how this was counterbalanced by optimism concerning trial outcomes. Finally, we present data that describe how levels of TO were sustained during the trial.

### Accounting for uncertainty: initial perceptions of trial design

*Research midwife O: See the big thing really for us has been the hospital staff being on board…They’ve been really really good. They’ve been watching out for patients so even when we’re not physically here they’re actually, you know, they’re helping us with recruitment. … ‘cause without them on board we’d not get anywhere with it.*

Because involving clinical staff in research, particularly those who work in highly pressurised environments, is not always straightforward, as Research midwife O commented, our interviewees were keen to reflect upon why they and their colleagues had decided to become involved in the Got-it trial. Their accounts revealed the existence of various degrees of uncertainty at both personal and local levels, which many described as influencing their engagement with the trial. Dr D, who had positive experiences of using the study drug in the past, still saw uncertainty about the trial outcomes as providing a rationale for their involvement with the research.

*Dr D: …it’s plausible that it might work. I’ve seen GTN used in other emergency situations in uterine inversion. We couldn’t get the uterus back in. We used GTN to relax the uterus. So I can see the physiology of why it’d work. I don’t know if it will though (laughs). I suppose that’s why we’re doing the study.*

Others, in contrast, expressed more scepticism about the use of GTN in this setting. As Dr E said: “if I’m honest, I don’t think it will [work]. I’m really intrigued and I’d love it if it did…But if I was gonna be a betting person, [I] would say that I think it’s not gonna show, it won’t…”.

There was evidence of scepticism and uncertainty coexisting at an individual or local (site) level without this having a deleterious effect upon trial delivery. Dr H, for example, reflected that even though the staff at their site were sceptical about trial outcomes, this did not necessarily put them off recruitment: “…the majority of the staff who are a little sceptical are still suitably opened minded that there’s still an uncertainty, that they’re willing to give it a go.”

However, only a small number of staff described themselves (or were described) as sceptical about the study. The majority of our interviewees said that initially, at least, they remained uncertain about trial outcomes.

*Dr A: I have given GTN to try and relax the cervix, whether it’s that mechanism… I don’t know for sure so I believe there is some effect with muscle and things so I can see that, you know, potentially it will, it could have an effect and therefore, I definitely think it’s worth trying. But I don’t think we know.*

This latter group talked positively about Got-it. Some praised the trial’s scientific rationale, like Dr J:

*Yeah, it’s definitely a good, good idea, and the thinking about behind it is fairly reasonable as well, in terms of using GTN to relax the uterus to deliver the placenta. Yeah, so I think it’s a really good trial.*

Others in this group extolled the need for, and thus the potential benefits of, the intervention.

*Clinical midwife C: But to be honest, I think the only incentive that I, or any midwife, would need is the possibility that it might work. And that you might be avoiding a trip to theatre — no one wants a trip to theatre, no one: the midwife doesn’t want a trip to theatre, the doctor doesn’t and the woman certainly doesn’t. So we’re all working towards the same ultimate goal, to get the placenta out without going to theatre. So for me, that’s all the incentive I need, I would do anything to get that placenta out without going to theatre.*

Many interviewees dealt with their uncertainty regarding the use of GTN in this clinical setting by adopting a hopeful attitude towards the trial. The interviews revealed that staffs’ TO about Got-it was fuelled by a number of situational factors. The first, as the next section demonstrates, was staffs’ familiarity with the study drug: GTN.

### Familiarity breeds therapeutic optimism: perceptions of the study drug

Many staff members commented that their prior knowledge about the way GTN works in the body made them feel more confident about becoming involved with this trial, because they felt that they understood the rationale underlying the study hypothesis.

*Dr E: I think it helps staff, or it brings up more questions I guess, ‘cause staff are thinking they know what the drug is. So they’re instantly thinking, well GTN does that. Ooh we’re doing the opposite to what we normally do with oxytocin we make everything contract, we’re getting everything to relax… It just makes it more accessible I guess, in the sense that people recognise the drug, so instantly can start to hypothesise what the rationale is for the trial, without reading anything.*

The fact that most staff had used GTN before and were aware of its effects and side effects gave them a feeling of security when recruiting participants and administering the intervention. As Dr F said: “The image I have of it is that it’s not particularly dangerous …I don’t see it as being a dangerous drug.” Indeed, most clinical staff said they regarded GTN as a safe intervention, even in this novel setting, a view that was influenced by using the drug in more familiar but notably different clinical contexts, such as for the treatment of angina.

Staff also described their previous experiences of using GTN as making them feel “more comfortable” using it in the trial (Dr C) and more confident about explaining drug-associated risks and benefits to potential participants.

*Dr H: It’s a lot easier actually to talk about a drug that you’ve got quite a lot of knowledge and experience and has been around for a long time than something new that all you’ve got is theoretical knowledge that you’ve been told.*

Familiarity with GTN, therefore, allowed staff to offset many of their uncertainties about the trial intervention. While they did not know what the outcome of the trial would be, the fact that they had used GTN before to beneficial effect influenced their views of the trial and their role in trial delivery. Previous experiences of using GTN were described as enabling staff to confidently weight the risks and benefits of study participation and allowing them to reach the conclusion that Got-it was a safe trial, with less uncertainty and fewer unknowns than trials which involve novel and untested treatments. Prior knowledge can thus be seen as promoting staff buy-in from the outset; because staff were familiar with GTN and perceived it as a relatively risk-free intervention, they were optimistic about the Got-it trial and happy to become involved in recruitment. However, familiarity with GTN was not the only situational factor that was reported as influencing optimism about Got-it. As the next section demonstrates, TO was also motivated by the fact that the trial potentially addresses what all staff identified as a pressing clinical need.

### Constructing therapeutic optimism: perceptions of clinical need

All interviewees talked about the impact GTN could have on peripartum care if the trial were to be successful, and these potential benefits clearly drove optimistic views of the trial.

*Research midwife M: I think the benefits of it [GTN], if it did work, would save such a lot of time, inconvenience, pain for the woman, distress and separation. And there are so many benefits I think the midwives are keen to do it. The way they say, ‘if it does that, and prevents all that, how good this will be’. And especially for the future, if it does work, being able to use in birth centres and community settings. Yeah the benefits of it will be really good if it did just have an effect.*

The fact that trial participation may prevent a woman from having to go to theatre to undergo what some described as a brutal and “degrading” (Clinical midwife N) procedure and be parted from their baby was described as the ultimate incentive for recruitment.

*Research midwife G: I think if it [GTN] can prove to work, then fantastic. It’s definitely worthwhile trying it and seeing, you know, where it comes from. Cause I think anything that can stop the women to have — a manual removal’s just awful. And I think when you’ve seen a few, it’s so barbaric you feel like you’re on a farm. Poor woman, watching them go through that. So I think anything that can be shown to stop that having to happen, then fantastic.*

Others noted that if GTN is shown to work in this context, then it would not only benefit women and their family, but also obstetrics more widely, because it would decrease staff workload and result in better targeting of clinical resources.

*Dr I: From the doctor’s point of view, you know, it’s obviously better for the woman as well, less risk and less medicalisation the day after. But it’s also less work. You can concentrate, focus on women who are in labour, or who may be sick or you know, other women.*

Got-it was seen as an easy trial to deliver, not only because the intervention is relatively straightforward to administer [17], but also because the staff identified a pressing clinical need for a drug that would enable women to deliver their placenta safely and simply. Clinical midwife N expressed her hope that the trial would demonstrate the efficacy of the intervention.

*Clinical midwife N: I hope it does… if it works it will be amazing because it will reduce these women going to theatre for such an invasive procedure …if it works it will be amazing because it will keep families together.*

Thus, TO was motivated by staff perceptions of clinical need, which in turn provoked a commitment to recruit women to the trial.

*Research midwife H: I think, with this trial in particular, because it is to do with retained placentas. And the midwife, you know, often does feel bad when they have to go to theatre. And you do question, could I have done anything different that would have changed the outcome. So by having some other option, rather than theatre, I think lots of midwives feel, you know, we really want to try this.*

However, as the next section demonstrates, TO did not remain constant throughout this trial. Indeed, there was evidence that for some staff members, and in some sites, TO was constantly shifting, and these fluctuations were described as potentially impacting on recruitment rates as the staff concerned engaged with or disengaged from the trial.

### Maintaining therapeutic optimism and sustaining trial recruitment

Therapeutic optimism is all about outcomes, not only hoped-for or desired outcomes, but actual eventualities. The staff interpreted outcomes for women in the trial — the delivery of a placenta in the delivery room following administration of the study drug or the manual removal of a placenta in theatre — as either the realisation or eradication of their hopes, respectively. There was evidence that staff were informally monitoring trial outcomes and that this influenced levels of TO. For example, Dr I reflected that it was a combination of their prior knowledge of GTN and witnessing GTN actually “working” in the trial that had increased their optimism about Got-it and led them to conclude that “it will show something.”

*I: Is it working so far?*

*Dr I: Yes I think so. And the staff think so. Because they think they can smell the drug. But obviously that’s just in their head. The Band 6 midwives have said as soon as the puffs are given it seems to come out. I think one midwife’s been involved in two cases, well we’ve only — done six or seven, so you know. But I think it will show something…And I think from what I’ve seen it seems to work. …I think, the way the drug works, it should — I’m hoping it’ll work. And I think it will. That’s just because of the drug characteristics and a little bit of what I’ve seen.*

Increasing levels of TO were seen to affect staff behaviour. Research staff described how the delivery of a placenta following randomisation had resulted in increased levels of optimism about Got-it at their site, which resulted in greater numbers of women recruited in the immediate aftermath.

*I: Do you feel that helps once there’s a success?*

*Dr H: When we had that one everybody was really excited and I think fairly shortly after that we randomised quite a lot more people.*

But not all randomisations result in successful outcomes, and staff went to great lengths to ensure levels of TO were maintained despite witnessing negative outcomes for women. For example, in some cases when women had gone to theatre for a manual removal after administration of the intervention, it was suggested that the outcome was nearly successful, because they had not required a “proper manual removal”, and it was speculated that, in these cases, women had nonetheless received the study drug.

*Research midwife A: In [site] there’s been seven [randomisations]. So far all our ladies have unfortunately gone to theatre. However, I think about three of them the placenta has been sitting in the vagina when they’ve got there. So, we would, well I — would assume that they possibly have had the drug rather than the placebo as opposed to the ladies that have gone on to need a proper manual removal, it suggests that they’ve possibly had the placebo.*

It was frequently observed that if a woman had needed surgical intervention, then it was more likely that she had been allocated to placebo. As Clinical midwife N commented: “I think she got placebo because it didn’t come out. I do think that she did get a placebo but we don’t know do we, so I don’t know.”

While successful outcomes and speculating about trial allocation may enable staff to maintain TO, if a centre witnessed a large run of manual removals following administration of the intervention, this could result in declining levels of TO.

*Research midwife I: I think we may have six or seven [recruits], and then it’s only one that’s been successful. …I’m not sure why, I don’t know the reason. They might all be placebos they’re getting. Or it may not work. It’s hard to say, isn’t it? But, we just feel a bit disheartened really. And it kind of drops the morale with the staff a little bit as well, when you just keep getting a bit of a negative result with it. And we think that staff might start to think, is there any point in doing the Got It trial. It’s not going to work … I just feel it would be nice if we got some positive results to kind of encourage staff a little bit more.*

A number of the research staff across the pilot sites said they worried that a lack of successful outcomes or an increasing number of adverse events in their site could negatively affect trial recruitment rates, as staff became less optimistic and more sceptical about trial outcomes. Although none of the staff interviewed at Site C reported engaging in selective recruitment practices, they were reported as adopting a more precautionary stance with regard to recruitment after a number of postpartum haemorrhages (PPH) occurred following administration of the study drug.

*Research midwife G: ‘Cause we’ve had quite a few PPHs. And obviously, we don’t know if it’s linked or not. But it’s put a lot of the midwives, and doctors [off] …when they talk, quite candidly, then they will be honest and just say, well it does put them off, because they don’t want to go in — a woman that’s quite nice and stable, and then they seem to take two puffs of this and they suddenly start bleeding and it becomes an emergency situation, rather than a calm situation that needs dealing with. It seems that, a few of them have had that repeated experience and it’s just really put them off.*

However, the research staff also suggested that declining TO (and recruitment rates) and a negative shift in levels of uncertainty at an individual/site level could be reversed by a run of successful outcomes, which, as they speculated, would “instil faith” in the trial and boost TO, and thus, stimulate recruitment. As Research midwife O said: *“*…we’ve had now four who haven’t worked, one that has. So if they just give me a few more that work I think to instil faith.”

Such comments highlight the complex relationship that exists between TO and uncertainty for staff. Research staff, in particular, acknowledged that TO or “faith” in the trial, was important for individual staff members to sustain the level of uncertainty, which they regard as necessary to continue to recruit women to the trial. Witnessing successful trial outcomes can be seen as important in this respect, because they appeared to directly affect staff perceptions of the trial, in the sense that a successful delivery was described as leading to increased optimism about the trial, which results in a motivation to recruit. A lack of success, particularly a run of visits to theatre or adverse events (Site C), was reported as leading to declining levels of TO and potentially negatively affecting levels of uncertainty, which resulted in staff emotionally and (potentially) physically disengaging from the trial, as they began to perceive the research as conflicting with their duties of care.

In summary, TO was reported as a dynamic phenomenon, as fluctuating at both an individual and site level during the pilot phase of Got-it. As noted above, these fluctuations in TO appear to be directly influenced by situational factors — recruiting staff members witnessing particular trial outcomes — and can be seen as important, because if levels of TO decline too far and too widely within a site, then, as discussed in the next section, staff may shift out of individual equipoise and trial recruitment may be negatively impacted.

## Discussion

This paper has reported the experiences of research and clinical staff involved in the delivery of a peripartum RCT in the UK: the Got-it trial. The data suggest that recruitment to Got-it could be indirectly influenced by shifting levels of TO about the trial held by staff members. It was observed that, while there was evidence of uncertainty about trial outcomes at a personal level, this uncertainty was balanced by the hope that the intervention might work and this TO motivated staff engagement with the trial. TO, in this instance, appeared to be influenced by a number of situational factors, i.e. staff members’ familiarity with the study drug and perceptions of clinical need, and was reinforced or undermined by witnessing the outcome of administering the intervention to their patients.

The interviews suggest staff were informally monitoring trial outcomes in individual cases to see whether the intervention was working or not, and this resulted in fluctuations in levels of TO. Indeed, there was evidence that staff not only continuously scrutinised individual outcomes at their site, but also re-framed negative outcomes to reinforce pre-existing levels of TO. This monitoring and reframing of trial outcomes can be seen as important, because it was observed that declining levels of TO have a negative impact on levels of uncertainty, resulting in greater scepticism, which in turn may negatively affect recruitment rates. Following a brief discussion of our data in the light of Jansens’s analysis of TO [6], we will consider the wider implications of TO for trial delivery, specifically, its relationship with individual equipoise and some of the ethical and practical implications of our analysis.

### The nature of TO in the Got-it trial

Jansen [6] argues that in order to (ethically) accommodate TO within research, we need to distinguish dispositional and situational optimism. While we did not investigate dispositional optimism in this study, our analysis of staff experiences of trial delivery suggests that their views of the trial and commitment to recruitment were, at least in part, underpinned by realistic situational TO. Our data suggest that staff’s situational TO involved a complex convergence of personal (knowledge and perceptions) and external (trial outcomes) factors that were particular to this situation.

First, staffs’ prior knowledge of the study drug and previous experiences of using it in clinical situations meant that they felt they could realistically appraise the trial hypothesis; they could see how GTN might plausibly work in this setting. Staffs’ familiarity with the trial intervention meant that, from the outset, they saw Got-it as a feasible, low-risk study, primarily because they perceived GTN as a tried and trusted intervention and also one which is easy for women to self-administer [17]. These staff did not just blindly hope for the best, like the parents entering their children into high-risk early-stage trials described by Woods and colleagues [2] or the staff [4] and patients [4, 13] involved in phase 1 trials. Rather, when considering whether to become involved with Got-it, they drew upon their pre-existing stock of knowledge of and experience with GTN, and this knowledge can be seen as underpinning their optimism about the trial outcomes.

Second, the data suggest that TO is grounded upon staffs’ perceptions of clinical need. The staff described Got-it as potentially addressing an unmet clinical need in their patients, and, therefore, they wanted the trial to work. In this sense, the staff’s views of Got-it are similar to those recounted by Miller, who argues that the optimism displayed by clinician-researchers involved in phase 1 trials serves as a form of affect management when no other therapeutic options are available and the clinical need is perceived as high [4]. The difference between the staff we interviewed and those described by Miller is that the former appeared to be realistic about potential trial outcomes, not least because TO was grounded in their knowledge of the study drug and on-going observations of trial outcomes, whereas the TO expressed by clinicians in phase 1 trials often involves TME and has been seen as unrealistic [4].

Third, the reports of fluctuating TO at individual and site levels confirm that the type of optimism observed in the Got-it trial is situational rather than dispositional [6]. TO in Got-it was influenced by witnessing the intervention apparently working or failing in individual cases, which often resulted in increased or decreased levels of TO. The fact that outcomes for women were reported as being so influential was not unexpected, as the effects of medical outcomes on levels of TO are frequently noted in clinical contexts, where it is observed that patients’ positive responses to treatment reinforce TO [1].

Finally, it can be argued that the TO displayed in staff accounts is realistic. Not only did their prior knowledge about GTN enable staff to make informed judgements about trial design, but also they described themselves and others as constantly reassessing their perceptions on the basis of their trial experiences. Thus, TO about the Got-it trial was explicitly grounded in staffs’ real experiences of trial delivery, not hypothetical speculations about its potential therapeutic benefits [2, 4]. Arguably, it is this balancing of hope and scepticism about the trial, in the context of their former and current experiences, that affects staff engagement with the Got-it trial and, ultimately, influences and affects recruitment practices. In the following section we will discuss how TO interacts with uncertainty, or individual equipoise, to potentially affect trial recruitment.

### The relationship between TO and individual equipoise: balancing hope and uncertainty

Before we discuss the role played by TO in counterbalancing scepticism about trial outcomes to sustain uncertainty, or individual equipoise, we need to distinguish different types of equipoise. Freedman argues that clinical rather than theoretical equipoise is needed if research is to be regarded as ethical [16]: “Clinical equipoise is defined as present or imminent controversy within the clinical community over the preferred treatment” ([16] p.141). He suggests that clinical equipoise is important from an ethical point of view, because it does not require individual researchers to be in a personal state of uncertainty in order to become involved in research.

Clinical equipoise is knowledge^3^ based, it involves balancing the objective evidence for and against different interventions and requires acknowledgment within the expert clinical community that there is sufficient uncertainty about which treatment should be used. In other words, clinical equipoise rests on a lack of consensus, or disagreement, within the expert clinical community about preferred treatment [16]. In contrast, when researchers describe themselves, or their local community, as being in equipoise what they are often referring to is a phenomenological state of uncertainty. Thus, individual and local [20] equipoise is not (only) based on the weighing of objective probabilities or scientific facts and/or an awareness of clinical equipoise, but also involves subjective perceptions of certainty and uncertainty. Individual equipoise can be seen as equally as important as clinical equipoise for some researchers because, arguably, it is their uncertainty about research outcomes which enables them to engage in research without feeling that they are compromising their clinical obligations [20, 21].

Individual equipoise may fluctuate throughout a trial, as individuals or the local community (e.g. trial site) begin to perceive trial outcomes as becoming more or less certain, and begin to favour one trial arm over another as a result [20, 21]. Once researchers, either at a personal or group level, believe that a particular trial outcome will or will not occur, they can be said to lack equipoise, and this can undermine the ethical nature of the trial if it influences the recruitment process [20, 22]. Indeed, in a recent study of a number of clinical trials that were experiencing recruitment difficulties, Donovan and colleagues report that a lack of individual equipoise regarding particular types of patients resulted in selective recruitment practices on the part of individual doctors, which biassed ascertainment in some of the trials they observed [23].

The impact of shifting equipoise on staffs’ intentions to recruit to trials may be explained by the fact that staff find certainty and uncertainty in clinical trials difficult to manage. Donovan et al. [23] and Snowdon [20] report the staff they studied as moving in and out of equipoise during the lifetime of a trial. In these studies, staff dealt with uncertainty by embracing it or dismissing it and opting for greater certainty displayed by personal [23] or site [20] preferences. In our study, interviewees’ involvement in recruitment appeared to have had less to do with levels of individual equipoise, or individual or group beliefs about the efficacy of the trial, than their hopes that the trial intervention would work and their patients would not have to undergo a surgical procedure. Indeed, it can be argued that the relationship between levels of TO and individual equipoise in the Got-it trial was crucial in determining staffs’ commitment to recruitment.

Our data suggest that the relationship between TO and uncertainty about trial outcomes, or individual equipoise, is complex. As noted above, individual equipoise can be seen as a dynamic phenomenon in which perceptions of certainty and uncertainty constantly shift during the lifetime of a trial [20]. We suggest that the point at which fluctuating levels of certainty-uncertainty compromise individuals’ obligations of care, and consequently threaten trial recruitment, is dependent on individuals’ TO. In this study TO, or a desire for the trial intervention to work, was frequently expressed by staff involved in trial delivery and, arguably, this TO was essential for sustaining levels of equipoise in staff members who had repeatedly witnessed negative trial outcomes. This was particularly evident in the sites where growing scepticism about the trial was observed amongst staff members; the research staff in these sites worried about staff’s disengagement with the trial, observing that staff need to maintain a certain degree of uncertainty about trial outcomes, i.e. to be in equipoise, to motivate them to keep recruiting in the face of seemingly negative outcomes. In other words, we would argue that TO was essential to recruitment in the Got-it study because it counterbalanced negative shifts in equipoise and thus allowed staff to retain the degree of uncertainty that they needed to continue recruiting to the trial.

This study suggests that TO and individual equipoise coexist in a finely balanced relationship within trials that permit on-going monitoring of individual outcomes. TO, like individual equipoise, is a dynamic phenomenon, which fluctuates throughout these trials and is contextually determined by individuals’ trial experiences. Thus, witnessing unsuccessful trial outcomes or adverse events may lead to declining levels of TO and disturbances in equipoise. If levels of TO should become too low, then individuals may no longer be able to sustain individual equipoise and, as a consequence, may disengage from the trial both emotionally and physically, and this could impact trial recruitment. Evidence of successful outcomes, on the other hand, maintains levels of TO in both individuals and study sites, which promotes engagement with the trial and sustains the level of uncertainty, or individual equipoise, that is deemed necessary for staff members to deliver the trial.

Of course, one thing we have not addressed is what might happen to trial recruitment if, in the light of their informal monitoring of trial outcomes, recruiting staff become unrealistically or over-optimistic about a trial intervention. While this was not an issue in Got-it, we can speculate that if staff only witness positive outcomes and/or are not exposed to negative outcomes or adverse events, then this may affect their levels of TO and individual equipoise. In this situation it is possible that staff will start to believe, rather than just hope, that the trial intervention works, and this assumption might have an impact on their recruitment practices. While this could increase a site’s recruitment rates, it will be at the expense of research participants’ autonomy and research standards, if staffs’ views of the trial influence the ways that they present the trial to potential participants. Moreover, over-optimism, or growing certainty about the efficacy of the trial intervention, could also cause staff to shift out of equipoise, which might result in them refusing to recruit their patients to RCTs, because they see this as compromising their duty of care. Such speculations suggest there is a need for further research that looks at the shifting relationships between evidence, equipoise and TO.

### Ethical implications

Like staff members involved in phase I trials [4], the staff we interviewed said they had become involved in Got-it because they had hoped the women they recruited would derive therapeutic benefit from their participation. Does it matter if staffs’ engagement in research is motivated by a desire for therapeutic benefit rather than scientific curiosity? It has been argued that the adoption of any therapeutic orientation to research can result in a blurring of the boundaries between clinical care and research, and that this may have negative ethical implications, particularly if it results in staff confusing the goals of research with treatment [4, 5], and conveying this to their patients [3]. As noted above, there was no evidence in this instance that the staffs’ TO about the use of GTN resulted in them “mis”understanding the nature of the activities in which they were engaged [3, 5], or that the TO displayed in their accounts was unrealistic [6]. Indeed, it was quite clear that staff were agreeing to recruit to Got-it because they hoped, but did not know/believe, that GTN might be effective in this setting. Moreover, as noted above, the fact that there was evidence of fluctuating TO at an individual and site level suggests that TO in this instance was firmly grounded in observed outcomes rather than unrealistic speculations or staff misestimating the outcomes.

Thus, following Jansen [6] and Horng and Grady [5], we would argue that holding an optimistic view of research is not ethically problematic per se; however, the extent to which researchers make their optimism explicit when recruiting trial participants is ethically important. We suggest that it would be ethically problematic for researchers to emphasise their hopes for a positive outcome during recruitment, because expressions of unsubstantiated TO, no matter how realistic, about the efficacy and utility of trial interventions, could bias participants’ perceptions and influence their decision-making and hence undermine informed consent.

### Practice implications

Before we look at how these findings could inform future trial recruitment practices, it is important to note that the impact of staffs’ observations of trial outcomes on levels of TO and the subsequent impact on recruitment may be related to the specific nature of the Got-it trial. Arguably, the fluctuations in levels of TO that we witnessed in Got-it, like the levels of shifting equipoise observed by Snowdon [20], were only possible because the design of the trial permitted on-going informal monitoring. As noted above, the Got-it trial took place in an emergency situation where staff and research participants made quick decisions, and trial outcomes were more or less immediately apparent, enabling staff to infer a causal link between administration of the intervention and outcomes. In other words, unlike other trials where recruiters may not deliver the intervention and observe outcomes, or where the primary endpoint may not occur for many years, in Got-it the recruiting staff received informal feedback about individual randomisations practically instantaneously. This meant that staff could get a feel for the trial findings while recruitment to the pilot was on-going, despite the fact that blinding was still in place, and adjust their levels of TO (and individual equipoise) to accommodate these observations. Thus, we can speculate that the role played by TO in trial recruitment may be particularly relevant in certain types of trials, namely those in which the trial outcomes are apparent to recruiters. Indeed, it can be argued that some of the issues raised in this paper may be heightened in research employing other types of trial design, for example, randomised open label or non-blinded trials, or complex interventions and non-CTIMP trials, for example, those where the intervention involves “talking” therapy versus standard care.

Given that there is an increasing number of trials taking place in which trial outcomes are known to recruiters during the recruitment period, our findings point to a need for on-going staff training and support throughout such trials to ensure that fluctuating levels of TO based upon staffs’ personal/lay observations do not “contaminate” trial recruitment and delivery practices. Recent research suggests that providing staff with training about the conceptual underpinning of trial delivery — clinical equipoise — and getting them to acknowledge their lack of individual equipoise or trial preferences facilitates trial delivery by enabling them to take a more objective stance towards trial recruitment and delivery [24]. Providing training throughout the lifetime of a trial that allows staff to reflect upon how their TO about the trial is sustained, maintained and reinforced by their trial and other experiences might similarly be helpful and ensure that TO does not bias recruitment rates.

### Limitations

The first limitation is that this study only recruited clinical staff who were involved in Got-it; therefore, it does not provide insight into the views of those who had declined to recruit to this trial. We can speculate that had we interviewed the staff members who did not want to become involved in Got-it, we might be presenting an even more rich and complex picture (see, for example, the study of Lumley et al. [22]). Second, bearing this in mind, it can be argued that the accounts we gathered involve a degree of self-presentation; it is possible that the staff we interviewed tailored their accounts of trial involvement to present themselves as responsible and morally upstanding individuals. However, while this may be the case, it is interesting to note that nearly everyone we interviewed decided to present their actions as primarily motivated by hope or TO. Third, these interviews were carried out during the first few months of the Got-it trial; one might, therefore, expect high levels of TO to be reported at this point. A prospective or longitudinal design involving interviews across the lifetime of a trial could be used to explore the possibility of wider fluctuations in TO and its effect on trial delivery at different time points. Finally, this study reports data from staff involved in one trial. As Snowdon [20] notes, staff feel and act very differently about different trials, even ones that take place in similar clinical settings; therefore, it is difficult to generalise from these results. This suggests that there is a need for further research into the influence of TO on researchers involved in different types of trials in different clinical settings.

## Conclusions

The influence of TO in clinical medicine has long been acknowledged, but the role played by TO in clinical research has been underspecified and under-researched until now. This paper looks at the function of TO in recruitment to a clinical trial. It is argued that the coexistence of hope and uncertainty, or TO and equipoise, may have implications for trial recruitment by enabling staff to sustain the level of individual equipoise needed to ethically deliver a clinical trial. It is observed that on-going, informal monitoring of trial outcomes may affect recruiting staffs’ levels of TO and, as a result, staff may require training and support so that fluctuating levels of TO do not negatively impact trial recruitment.

## Abbreviations

Got-it, Glyceryl trinitrate for retained placenta; GTN, glyceryl trinitrate; NICE, National Institute for Health and Clinical Excellence; RCT, randomised control trial; TM, Therapeutic misconception; TME, therapeutic misestimation; TO, therapeutic optimism.

## Footnotes

1 Although some authors argue that “hope” and “optimism” are different concepts (Bruhn [1]), they will be used interchangeably in this paper (cf. [2, 6, 12]).

2 http://www.isrctn.com/ISRCTN88609453?q=retained%20placenta&filters=&sort.

3 In arguing that clinical equipoise is based upon objective facts we are explicitly disregarding arguments about the social construction of knowledge, for example, Berger and Luckmann [25].
